# Two Outbreaks of Trichinellosis Linked to Consumption of Walrus Meat — Alaska, 2016–2017

**DOI:** 10.15585/mmwr.mm6626a3

**Published:** 2017-07-07

**Authors:** Yuri P. Springer, Shannon Casillas, Kathryn Helfrich, Deanna Mocan, Marscleite Smith, Gabriela Arriaga, Lyndsey Mixson, Louisa Castrodale, Joseph McLaughlin

**Affiliations:** ^1^ Section of Epidemiology, Alaska Division of Public Health; ^2^Epidemic Intelligence Service, CDC; ^3^Division of Parasitic Diseases and Malaria, Center for Global Health, CDC; ^4^Nome Public Health Center, Alaska; ^5^Public Health Associate Program, Office for State, Tribal, Local, and Territorial Support, CDC.

During 1975–2012, CDC surveillance identified 1,680 trichinellosis cases in the United States with implicated food items; among these cases, 1,219 were attributed to consumption of raw or pork products, and 461 were attributed to nonpork products. Although trichinellosis in the United States has historically been associated with consumption of pork, multiple nonporcine species of wild game also are competent hosts for *Trichinella* spp. and have been collectively implicated in the majority of trichinellosis cases since the late 1990s (*1*–*4*) (Figure 1). During July 2016–May 2017, the Alaska Division of Public Health (ADPH) investigated two outbreaks of trichinellosis in the Norton Sound region associated with consumption of raw or undercooked walrus (*Odobenus rosmarus*) meat; five cases were identified in each of the two outbreaks. These were the first multiple-case outbreaks of walrus-associated trichinellosis in Alaska since 1992 (Figure 2). Health care providers should inquire about consumption of commercially prepared and personally harvested meats when evaluating suspected trichinellosis cases, especially in areas where consumption of wild game is commonplace.

**FIGURE 1:**
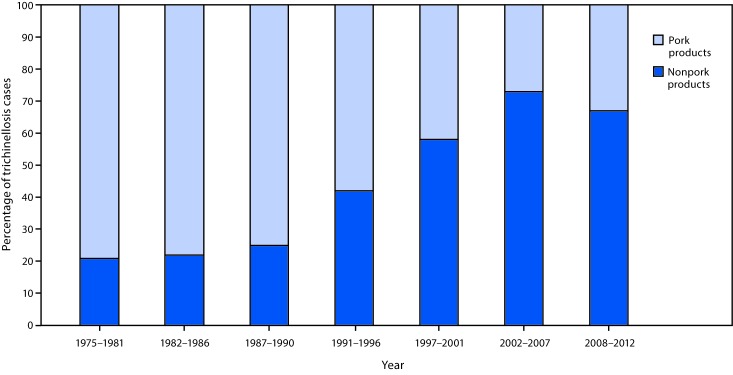
Percentage* of trichinellosis cases resulting from consumption of pork or nonpork products, by surveillance period^†^ among cases with a reported source (N = 1,680) — United States, 1975–2012 * Relative to the total number of cases in each surveillance period for which the food item implicated was identified. Pork products include both domestic pigs and wild boars. ^†^ CDC surveillance.

**FIGURE 2:**
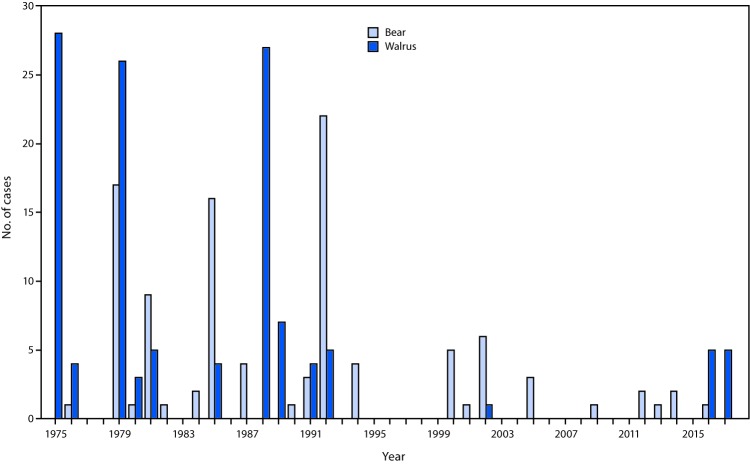
Number of cases (N = 227) of trichinellosis associated with consumption of bear* or walrus,^†^ by year — Alaska, 1975–2017^§^ * Bear includes 99 cases (44%) for which the patient reported consuming bear and four cases (2%) for which the patient reported consuming both bear and seal and a single implicated source of infection could not be identified. ^†^ Walrus includes 100 cases (44%) for which the patient reported consuming walrus and 24 cases (11%) for which the patient reported consuming both walrus and seal and a single implicated source of infection could not be identified. ^§^ As of July 1, 2017.

## First Outbreak

The index patient (patient A) was an adolescent female who reported severe lower extremity edema and pain, difficulty walking, a pruritic rash, weakness, fever, and myalgia beginning on August 15, 2016 (Table). She was evaluated at a village health clinic on August 31, 2016, and referred to the Alaska Native Medical Center in Anchorage, where she was admitted on September 8. Her adolescent brother (patient B) and father (patient C) accompanied her to the medical center, where they were also evaluated for symptoms of illness (Table). Blood tests indicated that all three patients had eosinophilia, a commonly observed sign of parasitic infection; two patients also had elevated creatine kinase levels, indicative of muscle inflammation (Table). All three patients reported having consumed raw or pan-fried (to “medium” doneness) walrus meat on approximately July 17. Serologic tests from both patients A and C were positive for *Trichinella* immunoglobulin G (IgG) by enzyme-linked immunosorbent assay (ELISA). The three patients received diagnoses of laboratory-confirmed (patients A and C) or probable (patient B) trichinellosis and were prescribed albendazole, an antiparasitic drug recommended for treatment of trichinellosis*; patient A also received prednisone.

**TABLE:** Clinical, laboratory, and epidemiologic characteristics of patients in two trichinellosis outbreaks associated with consumption of walrus meat — Alaska, July–September 2016 and April–May 2017

Patient	Relationship to index patient	Approximate date of walrus meat consumption	Approximate date of symptom onset	Symptoms	Eosinophils/*μ*L (%)*	Creatine kinase/*μ*L (reference)^†^	ELISA serology (case status)	Treatment
**First outbreak: July–September 2016**								
**A**	Self (index patient)	7/17/2016	8/15/2016	Lower extremity edema and pain, difficulty walking, rash, weakness, fever, myalgia	4,420 (17.6)	426 (26–192)	IgG+ (confirmed)	Albendazole, prednisone
**B**	Brother	7/17/2016	9/2/2016	Fever, myalgia	3,580 (20.7)	Not done	Not done (probable)	Albendazole
**C**	Father	7/17/2016	9/2/2016	Fever, myalgia, weakness, nausea, diarrhea	11,200 (50.4)	280 (36–174)	IgG+ (confirmed)	Albendazole
**D**	Aunt	7/31/2016	8/7/2016	Myalgia, fatigue, fever, chills, rash	2,080 (23.5)	3,391 (26–192)	IgG+ (confirmed)	Albendazole
**E**	Uncle	7/31/2016	8/7/2016	Myalgia, fatigue, nausea, diarrhea, weakness	4,930 (37.4)	3,300 (39–308)	Not done (probable)	Albendazole, prednisone
**Second outbreak: April–May 2017**								
**F**	Self (index patient)	4/25/2017	5/5/2017	Severe myalgia	6,850 (37.5)	2,150 (39–308)	IgG- (probable)	Albendazole, prednisone
**G**	Sister	4/25/2017	5/4/2017	Moderate myalgia	2,490 (22.0)	1,611 (26–192)	IgG+ (confirmed)	Albendazole
**H**	Mother	4/25/2017	NA	None reported	860 (11.0)	124 (26–192)	IgG+ (confirmed)	Albendazole
**I**	Neighbor (male friend, hunting partner)	4/25/2017	5/10/2017	Severe myalgia	2,330 (24.6)	854 (39–308)	IgG- (probable)	Albendazole
**J**	Neighbor (sister of patient I)	4/25/2017	5/10/2017	Moderate myalgia, facial edema	8,250 (51.3)	692 (26–192)	IgG- (probable)	Albendazole

**Abbreviations:** ELISA = Enzyme-linked immunosorbent assay; IgG = Immunoglobulin G, NA = not available.

* Reference range = 0–450/*μ*L (0.0%–5.0%).

^†^ Reference range varies according to patient’s age and sex.

On September 19, staff members of Norton Sound Regional Hospital in Nome reported two additional suspected trichinellosis cases from the same community, in the adult aunt (patient D) and uncle (patient E) of patient A. Both patients reported myalgia and fatigue beginning on approximately August 7, about 1 week after consuming raw walrus meat (Table). Blood tests confirmed that both patients had eosinophilia and elevated creatine kinase levels. ELISA testing identified IgG antibodies to *Trichinella* in patient D. Patients D and E received diagnoses of laboratory-confirmed and probable trichinellosis, respectively, and were treated with albendazole; patient E also received prednisone. Leftover walrus meat was not available to test for *Trichinella* larvae, and investigators could not determine when the walrus had been harvested, how widely associated meat had been shared, or whether all five patients had consumed meat from the same animal.

Interviews with patients and potentially exposed persons conducted by community health aides and staff members of the Nome Public Health Center did not identify any additional cases. Information regarding the health risks of consuming raw and undercooked meats was provided directly to the five patients. The risk reduction benefits of fully cooking meat according to U.S. Department of Agriculture recommendations for wild game (160°F [71°C], measured with a meat thermometer)^†^ before consumption, as well as trichinellosis symptoms, health effects, and methods of treatment were explained. The facts that the parasite cannot be reliably killed by smoking, drying, or fermenting meat, and that the arctic species *T. nativa* is freeze tolerant, were clarified. All five patients fully recovered. At the conclusion of the investigation, ADPH began developing a related public service announcement in collaboration with regional and village-based partners, with planned dissemination in multiple communities throughout northern and western Alaska during the 2017 spring walrus harvest.

## Second Outbreak

On May 12, 2017, as circulation of the public service announcement was beginning, ADPH was notified of another suspected case of walrus-related trichinellosis in a second Norton Sound coastal community, located <100 miles (<161 km) from the community where the first outbreak occurred (Table). Residents of both communities harvest walrus from the same hunting grounds in the northern Bering Sea. The index patient in the second outbreak (patient F) was an adult male who had been transported to Norton Sound Regional Hospital on May 12, 2017, after reporting severe muscle and joint pain. Blood tests revealed eosinophilia and elevated creatine kinase levels, but *Trichinella* IgG results by ELISA were negative; he received a diagnosis of probable trichinellosis and was prescribed albendazole and prednisone.

Interviews conducted by staff members of ADPH and the Nome Public Health Center identified four other suspected cases based on reported illness or likely exposure via a shared meal of undercooked walrus meat on approximately April 25, 2017. These patients were from two neighboring households that included members who hunted walrus together and shared the harvested meat. In the first household, the adult sister (patient G) and mother (patient H) of the index patient both had eosinophila, elevated creatine kinase levels, and positive *Trichinella* IgG results by ELISA; both patients were classified as having confirmed cases of trichinellosis. In the second household, an adult male friend and hunting partner of the index patient (patient I) and his adult sister (patient J) had eosinophilia and elevated creatine kinase levels, but negative results for *Trichinella* IgG by ELISA; both were classified as having probable cases. All four patients received treatment with albendazole. Given the high eosinophil counts and creatine kinase levels measured for the three patients with probable trichinellosis, it seems likely that these persons were infected but tested negative for *Trichinella* IgG by ELISA because the time elapsed between infection and testing was insufficient for a measurable humoral response.

The walrus consumed during the implicated meal in the second outbreak had been harvested and butchered by patients F and I during the previous 1–3 months, and the meat had been stored frozen in unlabeled bags in their respective household chest freezers. The meat was prepared by patient H, who reported that she boiled it for approximately 1 hour, after which the exterior was fully cooked, but the interior remained undercooked or raw, which was the desired result; interviewed persons reported that many community members prefer the taste and texture of undercooked or raw walrus meat to that of fully cooked meat.

No meat from the implicated meal was available for testing. Because of concern that some of the meat used to prepare the implicated meal (or from the same source animal) might still be present in bags in household chest freezers, a convenience sample of meat from 11 bags was collected. It was not possible to determine the number and identity of source animals represented in this sample, or whether the sample contained meat from the same animal consumed as part of the implicated meal. Samples were sent to CDC’s Division of Parasitic Diseases and Malaria’s service for laboratory testing. One sample was positive for larvae of *Trichinella* spp. using differential interference contrast microscopy and polymerase chain reaction (PCR) with primers specific to internally transcribed spacer regions 1 and 2. The parasite was determined to be *T. nativa* by sequencing PCR products.

All patients from the second outbreak made a full recovery, and no additional cases were identified. Information associated with the public service announcement, including risk reduction benefits of cooking meat fully and trichinellosis symptoms, health effects, and methods of treatment, was shared with the patients and the community at large through meetings with multiple groups.

## Discussion

Trichinellosis is a parasitic disease that results from consumption of raw or undercooked meat infected by roundworm species in the genus *Trichinella* (*5*). Early signs and symptoms occur 1–2 days after ingestion and include diarrhea, abdominal pain, fatigue, nausea, and vomiting. Systemic signs and symptoms, which typically occur 1–2 weeks after ingestion and last for 1–8 weeks, include facial and periorbital edema, fatigue, fever (remittent) and chills, headache, muscle soreness, pruritus (with or without a rash), nausea, difficulty coordinating movement, neurologic complications, and cardiopulmonary impairment.^§^

The significance of wild game species in the epidemiology of trichinellosis is apparent in Alaska. Among 241 trichinellosis cases reported in the state since 1975, 227 (94%) were attributed to consumption of nonporcine wild game, including ursid species (black bear [*Ursus americanus*], grizzly bear [*Ursus arctos*], and polar bear [*Ursus maritimus*]) and pinniped species (walrus and sea ice–associated seal species). Under the Marine Mammal Protection Act,^¶^ Alaska Natives may harvest marine mammals for subsistence purposes. Walruses, polar bears, and several sea ice–associated seal species are important for the nutritional, cultural, and economic well-being of many coastal communities in northern and western Alaska.

Since 1975, 100 (41%) of the 241 trichinellosis cases reported in Alaska have been associated with walrus meat and another 24 (10%) with walrus or seal meat. However, the frequency of walrus-associated trichinellosis in Alaska has declined sharply in recent years from an average of 6.3 cases per year (113 cases over 18 years) during 1975–1992, to an average of 0.5 cases per year (11 cases over 24.5 years) during 1993–2017 (as of July 1, 2017). Before the outbreaks described here, only one walrus-associated trichinellosis case had been reported in Alaska in the preceding 23 years. Reasons for this decline in incidence are unknown and might involve changes in parasite burden in walruses; the timing or location of walrus hunting; methods used to store, collect, handle, or prepare walrus meat for consumption; reporting practices among ill persons; and clinical testing methods or practices. These outbreaks underscore the importance of inquiring about consumption of commercially prepared and personally harvested meats, and about methods of meat preparation, when evaluating suspected trichinellosis cases, especially in areas where consumption of wild game in association with recreational or subsistence hunting is common.

These outbreaks also highlight the importance of culturally sensitive public health messaging. In areas where wild game species are harvested for subsistence, traditional methods of collecting, handling, preparing, storing, and consuming meat often have great cultural significance; however, some of these methods can be inconsistent with public health best practices. Rather than promoting or proscribing specific methods, public health messages that focus on communicating risks and explaining the manner and magnitude of risk reduction that can be achieved using different approaches (e.g., alternative methods of preparing meat for consumption) enable members of the target population to make informed decisions that integrate their traditional practices with their awareness and tolerance of risks.

**Summary**

**What is already known about this topic?**

Trichinellosis has historically been a disease most frequently associated with consumption of raw or undercooked pork products; however, nonporcine wild game species are now collectively implicated in the majority of trichinellosis cases in the United States.

**What is added by this report?**

During July 2016–May 2017, two outbreaks of trichinellosis (five cases each) associated with consumption of raw or undercooked walrus meat occurred in Alaska. Walrus meat has been implicated in half of all trichinellosis cases reported in Alaska since 1975, yet the frequency of walrus-associated trichinellosis in the state has declined in recent years for unknown reasons. The two recent outbreaks were the first associated with consumption of walrus meat since 2002 and the first multiple-case outbreaks since 1992.

**What are the implications for public health practice?**

Wild game consumption should be considered when evaluating suspected trichinellosis cases. Related public health messaging should be culturally sensitive to traditional methods of food handling and preparation.

## Footnotes

* https://www.cdc.gov/parasites/trichinellosis/health_professionals/index.html#tx.

†
https://www.aphis.usda.gov/vs/trichinae/docs/fact_sheet.htm .

§
https://www.cdc.gov/parasites/trichinellosis/disease.html .

¶
http://www.nmfs.noaa.gov/pr/laws/mmpa/mmpa_2015_revised_2017.pdf .
